# Molecular commensalism: how oral corynebacteria and their extracellular membrane vesicles shape microbiome interactions

**DOI:** 10.3389/froh.2024.1410786

**Published:** 2024-04-24

**Authors:** Jens Kreth, Emily Helliwell, Puthayalai Treerat, Justin Merritt

**Affiliations:** ^1^ Biomaterial and Biomedical Sciences, School of Dentistry, Oregon Health & Science University (OHSU), Portland, OR, United States; ^2^ Department of Molecular Microbiology and Immunology, School of Medicine, Oregon Health & Science University (OHSU), Portland, OR, United States; ^3^ Division of Infectious Diseases and International Health, Department of Medicine, University of Virginia, Charlottesville, VA, United States

**Keywords:** corynebacteria, streptococci, biofilm, oral-general health, commensalism

## Abstract

Historically, the study of microbe-associated diseases has focused primarily on pathogens, guided by Koch's postulates. This pathogen-centric view has provided a mechanistic understanding of disease etiology and microbial pathogenesis. However, next-generation sequencing approaches have revealed a far more nuanced view of the roles various microbes play in disease, highlighting the importance of microbial diversity beyond individual pathogens. This broader perspective acknowledges the roles of host and microbial communities in disease development and resistance. In particular, the concept of dysbiosis, especially within the oral cavity, has gained attention for explaining the emergence of complex polymicrobial diseases. Such diseases often stem from resident microbes rather than foreign pathogens, complicating their treatment and even clouding our understanding of disease etiology. Oral health is maintained through a delicate balance between commensal microbes and the host, with diseases like caries and periodontal disease arising from pathogenic perturbations of this balance. Commensal microbes, such as certain streptococci and *Corynebacterium spp.*, play crucial roles in maintaining oral health through mechanisms involving hydrogen peroxide production and membrane vesicle secretion, which can inhibit pathogenic species and modulate host immune responses. Recent research focused upon the mechanisms of molecular commensalism has expanded our understanding of these key functions of the commensal microbiome, demonstrating their central role in promoting oral health and preventing disease. These abilities represent a largely untapped reservoir of potential innovative strategies for disease prevention and management, emphasizing the need to bolster a symbiotic microbiome that inherently suppresses pathogenesis.

## Introduction

“…*Corynebacterium* emerges as the cornerstone of dental biofilms, forming the base of the community structure and interactions”—Ferrer, M.D. and A. Mira (1).

Traditionally, microbe-associated diseases have been investigated with a strong focus on pathogens (2). This approach is rooted in the paradigms established by Koch's postulates (3, 4). As a result, we have a robust understanding not only of the causative agents of diseases, but also of the molecular mechanisms supporting pathogenesis and the key processes occurring at the microbe-host interface. However, next-generation sequencing techniques developed in the last decades have redirected our understanding of the microbial role in disease development (5). The current view expands beyond a specific pathogen to an appreciation of the complex microbial diversity that can be present at sites of active disease, especially among chronic diseases of the human mucosae (6–8). While specific organisms undoubtedly play significant roles in such diseases, their mere presence is not always sufficient to cause disease (9). The host as well as the microbes surrounding pathogenic species also have their roles to play (10), either by supporting virulence or by failing to limit the overgrowth of tissue destructive organisms (6, 11). Hence, the concept of dysbiosis has been introduced in order to explain polymicrobial disease development, which is typically caused by resident microbes rather than by foreign invading pathogens (12). The oral cavity is one of the most common body sites affected by dysbiotic microbial communities (13).

Overall, the oral cavity provides an ecological niche for a highly diverse microbiome (14). While individual humans host between 150 and 300 different species in the oral biofilms of the mucosal surfaces, the tongue, the dentition, and the subgingival space, the collective oral microbiome of humanity is far more diverse (14, 15). It is believed that the resident microbes protect the host from pathogen invasion, while supporting mucosal tissue homeostasis and preventing excessive inflammation. Evidence indicates that the microbiome also supports proper immune system development (16). Despite this, the oral cavity hosts the two most common dysbiotic diseases of all of humanity, caries and periodontitis (17). Both are initiated by microbes that are natural residents of the oral cavity, which due to environmental factors, host behaviors, and host genetics, overrepresent virulence traits that trigger host tissue destruction (18). The result can lead to a positive feedback loop that favors the growth of normally low abundance tissue destructive species, ultimately triggering dysbiotic disease. The consequence of dysbiosis is that reversal to eubiosis is challenging, and polymicrobial diseases often become chronic with little chance of resolution (19).

In general, what turns normal microbial residents, a friendly consortium that works with our immune system to protect us from pathogens and diseases, into a harmful community that supports disease development? While we have a general understanding that certain human behaviors contribute to polymicrobial diseases, such as periodontal disease and ulcerative colitis, our comprehension of the molecular mechanisms within microbial communities remains poorly understood. This is perhaps due to the focus on only individual species within complex polymicrobial diseases. Since polymicrobial diseases are often resistant to treatment and seldom resolve, focusing solely on the disease state itself neglects earlier disease developmental steps. This might miss effective treatments options that could intervene with the disease in a much earlier and potentially less severe state. The question we need to ask is whether it is appropriate to focus on individual species contributions to dysbiotic pathogenesis. And does the answer lie in the active diseased state, or should we focus on much earlier events preceding disease development, maybe even focus on the healthy state? While this thought might be provocative, the reality is that, to date, neither caries nor periodontal disease can be cured. Therefore, the focus should be on disease prevention. Key to prevention is the fundamental knowledge of the processes that take place under healthy conditions, at the microbe-microbe and host-microbe intersection before a microbial community develops signs of disturbed homeostasis that can lead to dysbiosis.

## Molecular commensalism—key to a strong support of oral health

Commensal microbes are organisms that reside with their hosts, benefiting from them without causing harm, and in some cases, even actively participating in host-specific processes (16). However, the close relationship between hosts and their commensal microbiome, combined with an in depth understanding of their extensive communication network, reveals that commensals often indirectly benefit their hosts as well. Such benefits can arise through colonization resistance, where commensals compete with external pathogenic organisms, and through immunomodulatory roles that foster host-microbe homeostasis and support the development of a functional immune system (16). The sustenance of this homeostasis is intricately linked to interactions with commensal microbes, which can either synergistically foster the growth of a benign community or antagonistically to curb the proliferation of certain pathobionts. To describe the dynamics of commensal interactions more precisely, the concept of “molecular commensalism” has been introduced (16, 20). This term denotes the mechanistic processes that facilitate the proliferation of commensal communities, thereby preserving homeostasis and enhancing host health.

The investigation into molecular commensalism began by exploring the reciprocal relationship among commensal streptococci, such as *Streptococcus sanguinis* and *Streptococcus gordonii*, and the cariogenic *Streptococcus mutans*, contingent upon caries status (21). The commensal ability to maintain homeostasis was further confirmed in a more complex and ecological relevant six-species biofilm model, demonstrating that the absence of commensal *Streptococcus oralis* significantly increases *S. mutans* numbers (22). Our research has shown that hydrogen peroxide production by commensal streptococci can inhibit the growth of *S. mutans*, a process largely dependent upon pyruvate oxidase (SpxB) (23). This mechanism is pivotal for the competitive edge of oral streptococcal commensals. Beyond *S. mutans*, numerous pathobionts linked to caries and periodontal disease, such as *Actinomyces spp*. and *Porphyromonas gingivalis*, are particularly susceptible to hydrogen peroxide (24, 25). The gene encoding pyruvate oxidase is highly conserved and prevalent among oral streptococci. During the initial stages of biofilm formation, strains not coding for SpxB are in the minority. This is mainly due to the optimal conditions for pyruvate oxidase activity, which requires molecular oxygen. Therefore, pyruvate oxidase-dependent hydrogen peroxide production exemplifies an effective mechanism of molecular commensalism, due to its broad presence among oral streptococci (20, 26, 27). This mechanism presents a viable target for the manipulation of the oral microbiome to maintain oral homeostasis and promote health.

## Oral corynebacteria and their association with health

**Figure 1:**
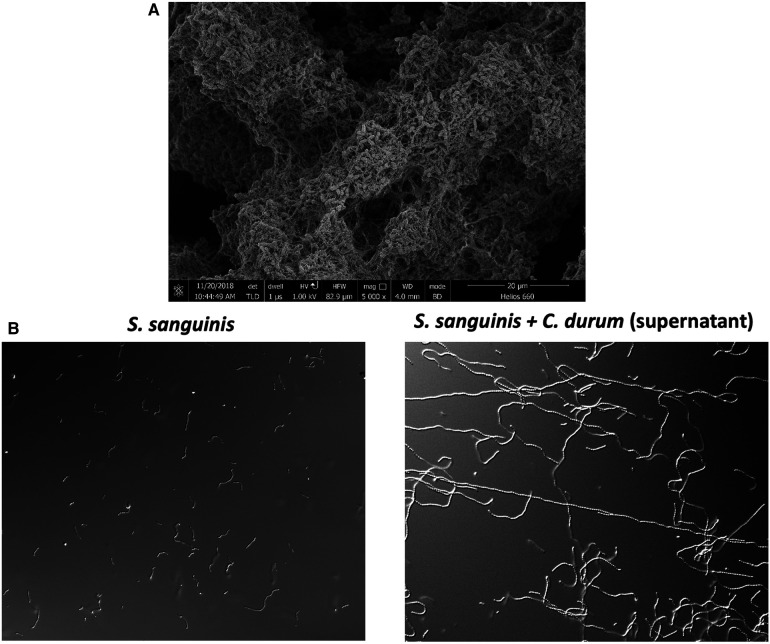
(**A**) Electron microscopy image of *C. durum* biofilm showing the production of extracellular matrix material. (**B**) *S. sanguinis* elongated chains after growth in *C. durum* supernatant.

While we laid the groundwork for understanding molecular commensalism with oral streptococci, our interest recently expanded to another prevalent bacterial species associated with oral health: *Corynebacterium durum*, a prolific biofilm and extracellular matrix producer (Figure 1A). This interest was sparked by a 2016 publication on oral microbial biogeography by Jessica Mark Welsh and colleagues (28). A detailed analysis of the spatial organization of the oral microbiome, using fluorescence *in situ* hybridization (FISH) combined with metagenomic sequence analysis of existing oral microbiome datasets, revealed a striking presence and arrangement of *Corynebacterium spp.* in supragingival plaque collected from healthy volunteers. Corynebacteria appeared to be among the most abundant species in the analyzed datasets, and its biogeographic localization within the supragingival biofilm enables species stratification along the filamentous growth of *Corynebacterium spp.* Consequently, distinct zones with unique environmental conditions seem to exist. Notably, the outer perimeter, with the distal tips of the filamentous corynebacteria, consists of corncob structures where streptococci mainly associate closely with corynebacteria. This zone is predicted to produce lactate, acetate, and hydrogen peroxide due to the more aerobic environment (28). The specific arrangement and association with diverse oral microbes suggest that *Corynebacterium spp.* are a key taxon in the development of supragingival plaque, which is also influenced by properties of the tooth surface its proximity to the gingival crevice where the corynebacterial filaments appear to attach (1, 28). Generally, oral corynebacteria are underrepresented in oral microbial research. For instance, a search for *Corynebacterium durum* in PubMed yields only 17 entries, mostly within the context of broader oral microbiome studies, with only a few focusing on this commensal at the molecular level. The Human Oral Microbiome Database V3.1 (HOMD) (29) catalogues 28 different corynebacterial species, but *C. durum* and *C. matruchotii* are among the most commonly isolated from human oral plaque biofilms. Numerous microbiome-based studies have confirmed a positive association between *C. durum*, *C. matruchotii*, and oral health. The prevalence of *C. matruchotii* significantly decreases in young children with diagnosed caries in permanent teeth (30). A negative correlation has also been observed between *C. matruchotii* and the occurrence of severe early childhood caries (s-ECC) (31). Moreover, in cohorts of children aged 6 to 12, *C. durum* was found to be enriched in non-caries samples (32). This health association is further supported by the observation that levels of *C. durum* and *C. matruchotii* increase following comprehensive caries treatment in children with s-ECC, suggesting the potential to restore an oral biofilm composition conducive to oral health, as speculated by the authors (33). Importantly, these beneficial species appear to be present in the oral cavity even under less favorable conditions, allowing for an overall recovery. Aside from its negative correlation with caries development, corynebacteria also appear to be less abundant in periodontitis (34, 35). Interestingly, analysis has shown *C. durum* to be associated with periodontal health and has also identified a positive correlation between the presence of *C. durum* and specialized pro-resolving lipid mediators (SPMs), which are crucial for resolving periodontal inflammation and include molecules such as lipoxins, resolvins, protectins, and maresins (36). Whether this correlation is causal remains to be determined. However, *C. durum* secretes several fatty acids with anti-inflammatory potential (37), suggesting that commensal corynebacteria may collaborate with oral mucosal host cells to promote periodontal health. Consequently, the *Treponema* to *Corynebacterium* ratio is being discussed as a novel microbial indicator of periodontitis (38). Overall, corynebacteria seem to have a strong association with oral health and might also actively orchestrate health promoting activities as we discuss below.

## The role of membrane vesicle in the oral corynebacterial support of the commensal community

The biogeographical study mentioned previously (28) spawned a new research interest within our group. Initially, our interest was in antagonistic activities that could inhibit pathobionts from becoming dominant (23). However, our investigation into oral corynebacteria enabled us to explore two crucial questions: (1) What are the synergistic activities among oral microbes that promote oral health? Notably, corynebacteria seem to prefer associating with hydrogen peroxide-producing streptococci; and (2) What are the molecular determinants of human oral microbial biogeography? In other words, why are some species found only in specific locations associated with certain species, but not in others? Since beginning our work on oral corynebacteria in 2016, we have developed significant insights into both questions (37, 39–41). A key finding was a unique interaction between *C. durum* and *S. sanguinis* that was not observed among various other oral streptococci: *S. sanguinis*, when co-cultured with *C. durum*, would grow into exceptionally long chains (Figure 1B). This growth phenotype seemed unusual because, in monoculture, *S. sanguinis* cell chains typically consist of fewer than ten cells. However, the *C. durum*-induced chain elongation phenotype results in chains extending more than tenfold (37). Intriguingly, *S. sanguinis* grown in saliva also exhibits this elongated chain phenotype (37), suggesting that co-culturing *C. durum* with *S. sanguinis* more accurately replicates the *in vivo* environment. After confirming that the induction of the chain elongation phenotype does not require physical interaction between the two species, we identified several fatty acids in the supernatant of *C. durum* cultures, including oleic acid, stearic acid, and palmitic acid. When these fatty acids were reconstituted *in vitro* and added to *S. sanguinis* growth medium, they reproduced the chain elongation phenotype. The carrier for the fatty acids secreted by *C. durum* was identified as extracellular membrane vesicles, which is an emerging research area, especially among Gram-positive bacteria (37). The long-standing assumption was that membrane vesicle production was not possible for Gram-positive bacteria due to the thick peptidoglycan layer of the cell wall (42, 43). The significance of the nano-sized, spherical, bi-layered extracellular membrane vesicles became apparent from their multitude of distinct biological activities, including cell-to-cell communication, biofilm formation, host cell interactions, antibiotic resistance, and long-distance signaling. The capacity for long-distance signaling is of particular importance (42). Extracellular membrane vesicles can elicit host immune responses similar to those triggered by bacterial cells and they can be internalized (44), potentially affecting cellular processes. Due to their compact nano-size, these vesicles can easily and safely travel to distant sites, potentially affecting tissues and microbes beyond the immediate vicinity of the vesicle-producing microbe. The fatty acid content of *C. durum* extracellular membrane vesicles is influenced by the carbon source used for growth (37), suggesting that corynebacteria modify their vesicle content based on environmental conditions and hinting at a controlled genetic process influencing vesicle biogenesis.

We further investigated how *C. durum* extracellular membrane vesicles, and *C. durum* in general, determine interactions with *S. sanguinis* to establish their role in molecular commensalism. Besides chain elongation, *C. durum* had several other effects on *S. sanguinis* that influenced overall fitness. Notably, the survival rate of *S. sanguinis* increased significantly after overnight culture with *C. durum*. Surprisingly, *S. sanguinis* also appeared more resistant to macrophage uptake, a trait independent of the chain elongation phenotype but dependent upon co-incubation, supporting a broader relationship between both species (37, 41). We also demonstrated that *S. sanguinis* pili are absent when grown in *C. durum* supernatant (41). Surface pili play a wide range of roles in microbial biology, including biofilm formation and surface attachment. *S. sanguinis* can translocate via twitching motility, another pilus-mediated function, which is abolished in the presence of *C. durum (*41). Our interpretation is that *C. durum* deliberately interferes with *S. sanguinis* pili production to prevent *S. sanguinis* from physically dissociating from their partnership, which could be seen as a potential mechanism of niche occupation determining oral microbial biogeography.

While our experimental discoveries were made *in vitro*, the close relationship between *C. durum* and *S. sanguinis* observed through chain elongation and intermingling is reminiscent of the corn-cob formation between corynebacteria and streptococci observed in native plaque samples (28). Although a direct interaction between *S. sanguinis* and *C. durum* in plaque samples has not yet been demonstrated, the fact that *S. sanguinis* forms much longer chains in saliva is suggestive. Additionally, while the observed chain elongation phenotype is thus far unique to *S. sanguinis*, *C. matruchotii* can also produce fatty acid-loaded membrane vesicles that induce chain elongation in *S. sanguinis* (unpublished). Thus, the *in vivo* partnership between corynebacteria and streptococci, in general, might be influenced, at least partially, by fatty acids and membrane vesicles. The potential benefit of an *in vivo* association of both species is significant; both are found in high abundance in healthy subject plaque samples, suggesting a protective function for the host. The observed stratification *in vivo*, with streptococci located distally to the tooth attachment site of corynebacteria, would ensure proper generation of hydrogen peroxide due to oxygen access and could interfere with caries and periodontal disease associated species that are more susceptible to hydrogen peroxide.

The implications of this relationship extend significantly and might also involve the host directly. Fatty acids are known for their anti-inflammatory properties (45, 46). We previously reported that biofilms of *C. durum* do not trigger strong responses of IL-6 and IL-8 in oral mucosal cell lines (39). This observation, while speculative, suggests that the minimal reactive nature towards host epithelial cells, along with the decreased macrophage uptake of *S. sanguinis (*37), could act as a protective mechanism for oral health, perhaps via anti-inflammatory mechanisms. Essentially, while *S. sanguinis* triggers the production of IL-6 and IL-8 in host epithelial cells, thereby keeping the host cells on alert for potential threats, the lower likelihood of being engulfed by macrophages reduces the chance of triggering a stronger inflammatory response, which would result in the release of inflammatory mediators by macrophages. This would maintain a low level of immunological alertness, preparing the host for potential invasions by pathogens or an increase in virulence of pathobionts. However, this hypothesis warrants further research for validation.

## Potential other health supporting roles of oral commensal corynebacteria

Dr. Hwang's group at Woosuk University, Republic of Korea, suggests that *C. durum* may play a wider role in supporting health, extending its benefits to the host (47, 48). Extracts from *C. durum* supernatants appear to be a rich source of secreted metabolites, several of which have been extracted and shown to possess properties that extend lifespan, as demonstrated in a *Caenorhabditis elegans* lifespan assay (47), and exhibit anti-cancer activities in various human cancer cell lines (48). The anticancer activity was also demonstrated for *C. matruchotii*, which in combination with another periodontal-health associated species, *Neisseria sicca*, is able to reduce tumor growth in a murine SCC-7 tumor-bearing model (49). Although it remains unclear if these metabolites and anticancer activities are associated with extracellular membrane vesicles, the findings indicate that commensal human-associated corynebacteria may have evolved to protect their host in diverse ways. Notably, nasal strains of *Corynebacterium spp.* are found more commonly in children who do not carry *Streptococcus pneumoniae*, a potentially life-threatening pathogen for young children (50). Specifically, one isolated strain, *C. accolens*, can release free fatty acids through a specific lipase, LipS1, from triacylglycerols on the human skin surface (50). Thus, human corynebacteria may influence not only the oral and nasal microbiome but potentially the overall human microbiome as well.

Overall, there are numerous studies highlighting the health-related benefits of (oral) corynebacteria, a species that has not been thoroughly explored but shows potential to be a beneficial commensal organism. This potential can be harnessed by enhancing its interaction with other commensal species, especially oral streptococci, and by investigating the mechanisms of oral commensalism to fully understand how corynebacteria contribute to health.

## Concluding remarks

Identifying the mechanisms of molecular commensalism will provide us with critical insights into the development of health-associated communities. Once we understand these processes, we can identify ways to bolster the commensal traits of certain species, thereby offering a protective measure that could redirect early changes in microbial communities. We have already established the feasibility of such an approach by augmenting hydrogen peroxide production. In a mouse model of colonization, supplementing the drinking water with magnesium significantly increased the colonization of the commensal bacterium *S. gordonii* compared to the control group lacking magnesium supplementation (51). This effect was mechanistically linked to the magnesium-dependent increase in oral streptococcal hydrogen peroxide production (51).

*Corynebacterium ssp.* are quite ubiquitous colonizers of humans and major health benefits have been already associated with a few investigated examples. Overall, we know very little about them. Membrane vesicles isolated from oral corynebacteria (and other health-associated microbial species) appear to hold promising potential for supporting molecular commensalism. They are easy to purify, retain their biological activity even after prolonged storage, and lack any replicative potential that would be associated with the bacterial cell itself, making them ideal carriers for bioactive molecules. However, the examples described here only represent a nascent understanding of a largely unexplored world of interspecies interactions in the healthy (oral) microbiome. With each new exploration, we expect to uncover additional facets of molecular commensalism.

## Data Availability

The original contributions presented in the study are included in the article/Supplementary Material, further inquiries can be directed to the corresponding author.
